# Supplementary material for: DNA synthesis for true random number generation
Source: Nat Commun. 2020 Nov 18;11:5869. doi: 10.1038/s41467-020-19757-y (PMC7675991; doi:10.1038/s41467-020-19757-y)
Supplement: Supplementary file 1 — Supplementary Information [file 41467_2020_19757_MOESM1_ESM.pdf]

Supplementary Information

Supplementary Information 1: Post-processing DNA pool confinement

The procedure for processing sequenced DNA to eliminate erroneous sequences, and to prevent primer nucleotides from being misread as random nucleotide is as follows: Initially, each DNA strand is searched for a 16-nucleotide sequence of the adapter. All strands containing this sequence (thus, containing the correct adapter), are shortened to 69 nucleotides. All other sequences were discarded. Further, all 69-nucleotide strands were searched for the first nine nucleotides of the adapter. This time, all sequences still containing this part of the adapter were discarded. All other sequences were cut to 60-nucleotide strands. This procedure was set in place to ensure the absence of any adapter nucleotides being incorporated in the desired random sequence. As we are solely interested in the randomly synthesized nucleotides, any constancy due to adapters could disturb the randomness results.

Supplementary Figure 1a shows the location of primer regions and the desired random sequence, as designed. The morphology of the DNA is shown for two cases: the ideal case, where no errors have occurred during synthesis and sequencing of DNA, and the actual case, where errors have occurred. As can be seen, errors can lead to shortened DNA sequences, in which case nucleotides of the primer region may get misread as part of the random region. This, in turn, would make the random region “less” random.

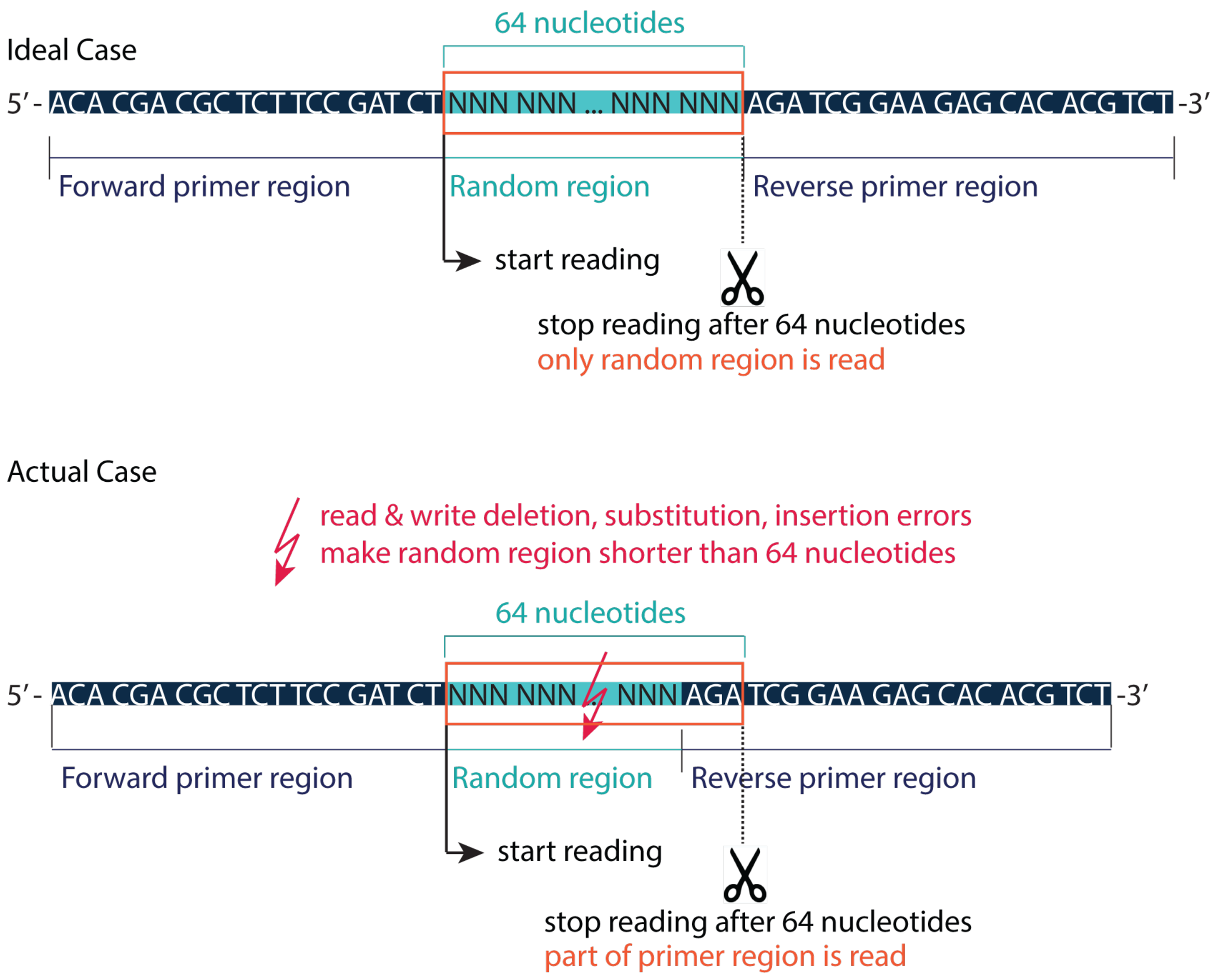

Supplementary Figure 1a: DNA Processing*. Due to synthesis and sequencing errors, DNA strands may be shorter than 64 nucleotides. Thus, when reading 64 nucleotides, part of the adapter region (which is constant and not random) could unintentionally be read. This induces additional bias into the data. The following procedure has thus been adapted to ensure the absence of adapter regions in data: 1) Find all sequences containing the adapter and include these sequences in the new pool. 2) Shorten these sequences to 69 nucleotides. 3) Find all sequences still containing the first nine nucleotides of the adapter and exclude those from the pool. 4) Shorten all sequences left in the pool to 60 nucleotides.*

An illustration of the exact procedure of sequence shortening can be found in Supplementary Figure 1b, which begins procedure explanation starting with the pool from which sequences not containing the correct 16 nucleotides of the adapter have been discarded. This procedure

illustrates the reasoning why sequences are shortened to, first, 69 nucleotides, and then, 60 nucleotides.

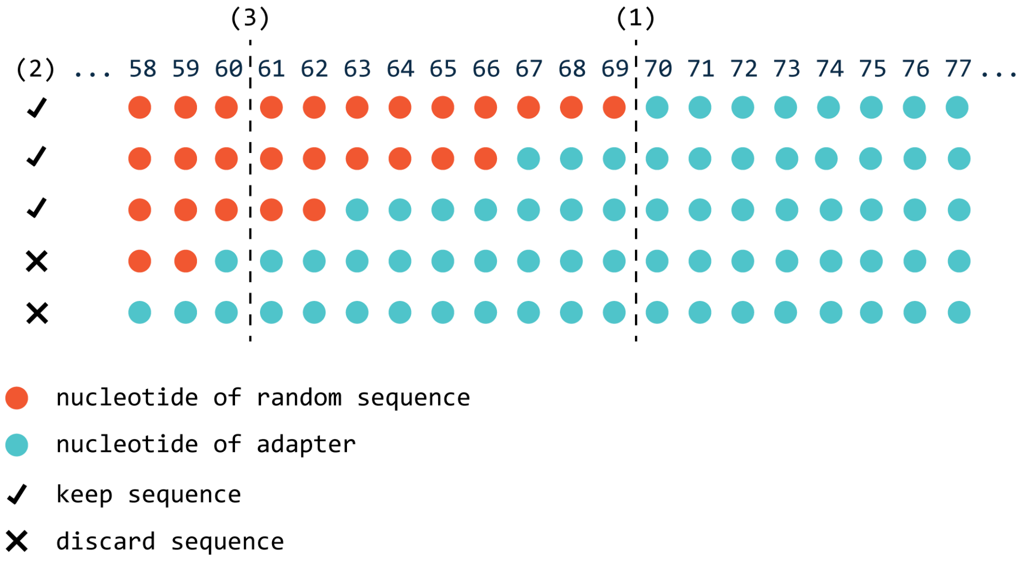

(1) cut all sequences containing 16 nucleotides of adapter at 69 nucleotides
(2) search for sequences containing 9 adapter nucleotides and delete those sequences
(3) shorten all other sequences to 60 nucleotides

Supplementary Figure 1b: Selecting error-free sequences. *Previously to this, the pool of DNA sequences has been searched for sequences containing 16 nucleotides of the adapter (AGA TCG GAA GAG CAC A). Sequences not containing these 16 nucleotides of adapter were discarded from the pool.*

Supplementary Information 2: NIST statistical test suite evaluation of raw bit stream of Microsynth synthesis 1

Supplementary Table 1: NIST statistical tests performed on raw synthesized DNA (cut to 60 nucleotides), mapped to bits using the scheme A→ 0, C → 0, T → 1, G → 1. For each test, 56 bit streams containing 1096 bits were tested

** for the rank test, 56 bit streams containing 100,000 bits each were tested*

**NIST statistical test**	**P-value**	**Proportion**	**Result**
Frequency	0	0/56	Fail
Block frequency	0	0/56	Fail
Cumulative sums	0	0/56	Fail
Runs	0	1/56	Fail
Longest run of ones	0	1/56	Fail
Rank*	0.534146	56/56	Pass
Discrete Fourier transform	0	53/56	Pass
Approximate entropy	0	56/56	Pass
Serial	0.000439	50/56	Fail

The first test is called the frequency test and evaluates the proportion of zeros and ones in the stream of bits. In a truly random sequence, the expected fraction of zeros and ones is ½. This test assesses how close the actual fraction is to the expected value by assigning zeros and ones to the values -1 and 1 and calculating the cumulative sum. Secondly, the block frequency test is a variation of the frequency test, where the proportion of ones and zeros is calculated within smaller blocks. We chose the block size to be $M = 128$. Third, the

cumulative sums test gives an evaluation determining if the stream contains “too many ones” or “too many zeros” as well as indicating if ones and zeros are intermixed too evenly. The fourth test is called runs test. The purpose of the runs test is to evaluate for uninterrupted sequences of zeros and ones. A run therefore is defined as a segment of one bit, being bound by the opposite bit on either side. The result of runs of various lengths is then compared to the expected distribution of run-lengths. Fifth, the longest runs of ones test is similar to the runs test with the difference that here the bit streams are divided into smaller blocks (here, a block consists of 8 bits), which are then evaluated for the longest runs of ones. The results are compared to the expected lengths of runs of ones of a truly random sequence. Sixth, the rank test focuses on disjoint sub-matrices of the bit stream. It checks for linear dependencies among fixed length substrings of the input bit stream. Seventh, the discrete Fourier transform test, tests for periodic features in the bit stream. Such features may include repetitive patters that are very similar to one another and would indicate a deviation from randomness. Test number eight is the approximate entropy test, which compares the frequency of all possible overlapping bit block patterns against the expected result. The test primarily focuses on patterns within the bit stream. The block length chosen for this experiment was 5 bits. The ninth and last test used to evaluate for randomness is the serial test. It also focuses on the recognition and existence of pattern by evaluating the uniformity of patterns’ distributions. For the serial test, a block length of 16 bits was chosen.

Supplementary Information 3: Robustness of NIST statistical test suite evaluation

We have performed a series of randomness evaluations using NIST statistical test suite, in which the robustness of the system was assessed with different technical parameters. We investigated different numbers of bit streams as well as different lengths of bit streams and observed the following: With 56 bit streams, each of length 10,000 bits, all tests pass. The shorter the bit stream length tested, the worse the Rank test performs and the longer the bit stream length tested, the worse the Runs test performs (with the performance of all other tests being similar in all cases). When increasing the number of bit streams investigated, the Runs test performance decreases. At high bit stream numbers and bit stream lengths, the Discrete Fourier Transform test starts showing negative results. All other tests show very robust evaluation performance at all values of bit stream length and number of bit streams tested.

Supplementary Table 2: Robustness of NIST statistical tests performed on Von Neumann processed bit streams, mapped to bits using the scheme A→ 0, C → 0, T → 1, G → 1.

**Number of bit streams**	56	56	56	100	100	100
**Length of bit streams**	50,000	10,000	1,096	50,000	10,000	1,096
**Tests failing**	Runs	All tests pass	Rank	Runs	Runs close to failing	Rank, Discrete Fourier transform close to failing

Supplementary Information 4: Table showing efficiency of Von Neumann de-biasing procedure

Mapping scheme A: A→ 0, C → 0, T → 1, G → 1

Mapping scheme B: A→ 0, C → 1, T → 0, G → 1

Mapping scheme C: A→ 0, C → 1, T → 1, G → 0

Mapping scheme D: A→ 00, C → 11, T → 10, G → 01

Supplementary Table 3: Efficiency of Von Neumann de-biasing procedure for different mapping schemes.

Synthesis Pool	Mapping Scheme	Von Neumann De-Biasing Efficiency [%]
Microsynth, synthesis 1	A	23.7
Microsynth, synthesis 2	A	23.3
Eurofins Genomics	A	23.6
Microsynth, synthesis 1	B	24.4
Microsynth, synthesis 1	C	25.0
Microsynth, synthesis 1	D	7.0

Supplementary Information 5: NIST statistical test suite evaluation of bit streams of Microsynth synthesis 2 (mapping A), Eurofins Genomics (mapping A), and Microsynth synthesis 1 (mapping B, C and D)

Mapping scheme A: A→ 0, C → 0, T → 1, G → 1

Mapping scheme B: A→ 0, C → 1, T → 0, G → 1

Mapping scheme C: A→ 0, C → 1, T → 1, G → 0

Mapping scheme D: A→ 00, C → 11, T → 10, G → 01

Microsynth synthesis 2, mapping scheme A (A→ 0, C → 0, T → 1, G → 1).

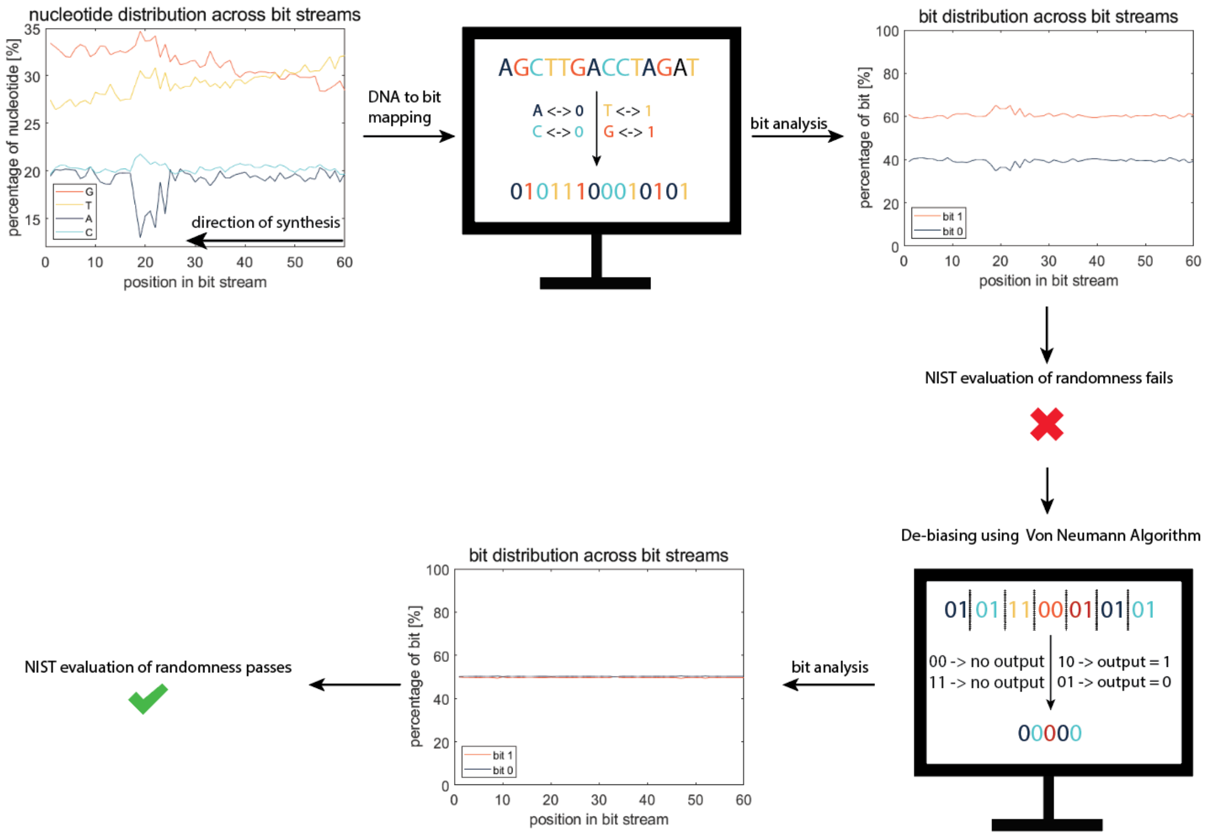

Supplementary Figure 2a: Microsynth synthesis 2, mapping scheme A (A→ 0, C → 0, T → 1, G → 1). *Data analysis procedure showing de-biasing effect of the Von Neumann algorithm.*

Supplementary Table 4: NIST statistical test evaluation of Microsynth synthesis 2, mapping scheme A (A→ 0, C → 0, T → 1, G → 1), after de-biasing.

**NIST statistical test after de-biasing**	**P-value**	**Proportion**	**Result**
Frequency	0.058984	56/56	Pass
Block frequency	0.075719	56/56	Pass
Runs	0.971699	55/56	Pass
Longest runs of ones	0.137282	55/56	Pass
Cumulative sums	0.699313	56/56	Pass
Rank*	0.779188	55/56	Pass
Discrete Fourier transform	0.000145	53/56	Pass
Approximate entropy	0.213309	56/56	Pass
Serial	0.000439	56/56	Pass

Eurofins Genomics synthesis, mapping scheme A (A→ 0, C → 0, T → 1, G → 1).

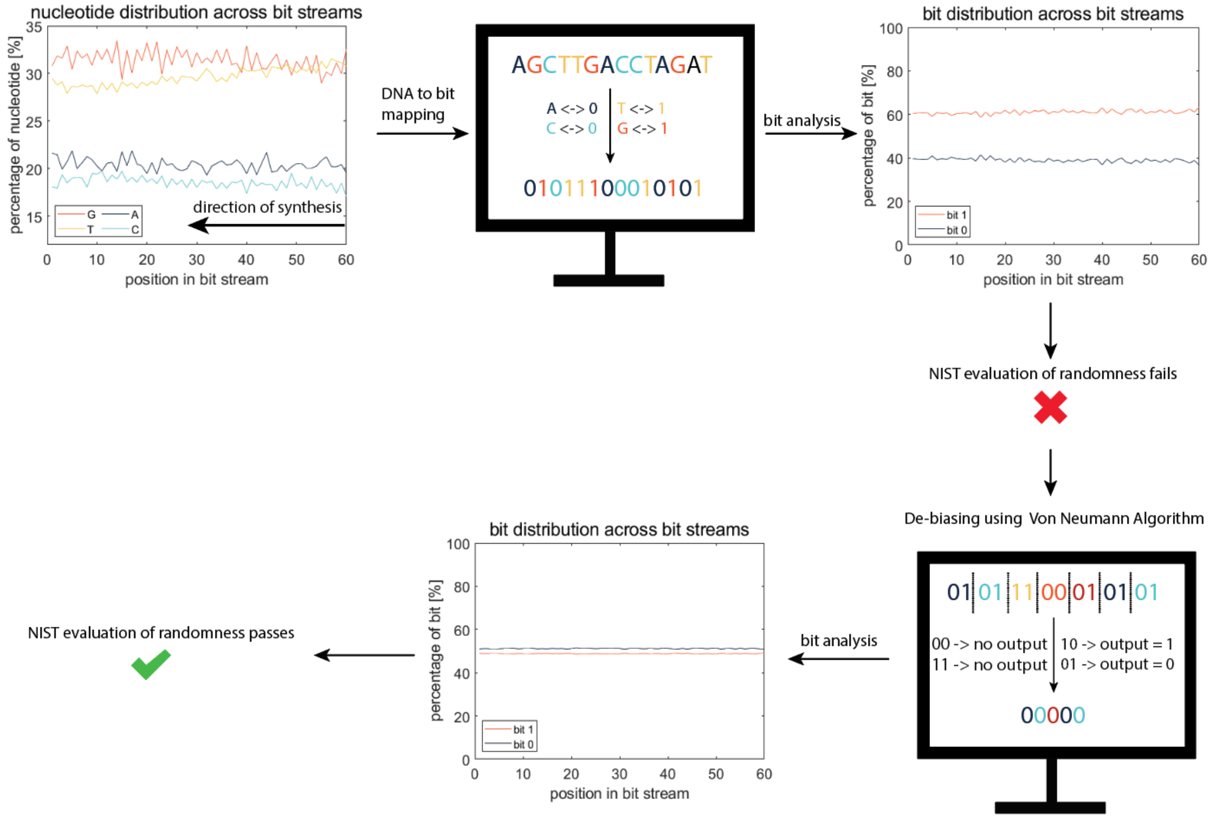

Supplementary Figure 2b: Eurofins Genomics synthesis, mapping scheme A (A→ 0, C → 0, T → 1, G → 1)*. Data analysis procedure showing de-biasing effect of the Von Neumann algorithm.*

Supplementary Table 5: NIST statistical test evaluation of Eurofins Genomics synthesis, mapping scheme A (A→ 0, C → 0, T → 1, G → 1), after de-biasing.

**NIST statistical test after de-biasing**	**P-value**	**Proportion**	**Result**
Frequency	0.006661	55/56	Pass
Block frequency	0.455937	56/56	Pass
Runs	0.085587	56/56	Pass
Longest runs of ones	0.699313	54/56	Pass
Cumulative sums	0.013569	55/56	Pass
Rank*	0.085587	56/56	Pass
Discrete Fourier transform	0.002374	54/56	Pass
Approximate entropy	0.350485	55/56	Pass
Serial	0.010237	56/56	Pass

Microsynth synthesis 1, mapping scheme B (A→ 0, C → 1, T → 0, G → 1).

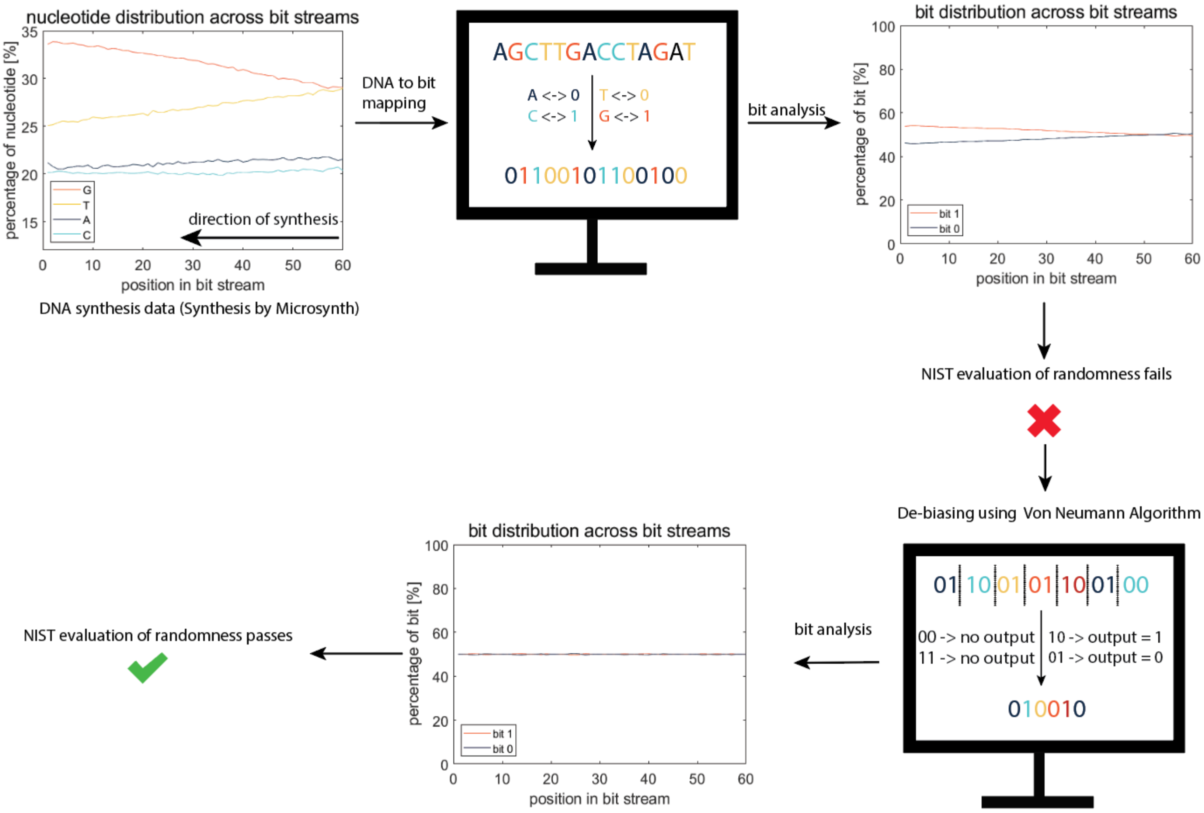

Supplementary Figure 2c: Microsynth synthesis 1, mapping scheme B (A→ 0, C → 1, T → 0, G → 1). *Data analysis procedure showing de-biasing effect of the Von Neumann algorithm.*

Supplementary Table 6: NIST statistical test evaluation of Microsynth synthesis 1, mapping scheme B (A→ 0, C → 1, T → 0, G → 1), before de-biasing.

**NIST statistical test**<br><br>**Before de-biasing**	**P-value**	**Proportion**	**Result**
Frequency	0.000233	54/56	Pass
Block frequency	0.000013	54/56	Pass
Runs	0.075719	54/56	Pass
Longest runs of ones	0.000700	51/56	Fail
Cumulative sums	0.001757	55/56	Pass
Rank*	0.534146	55/56	Pass
Discrete Fourier transform	0.000009	56/56	Pass
Approximate entropy	0.213309	55/56	Pass
Serial	0.066882	56/56	Pass

Supplementary Table 7: NIST statistical test evaluation of Microsynth synthesis 1, mapping scheme B (A→ 0, C → 1, T → 0, G → 1), after de-biasing.

**NIST statistical test After de-biasing**	**P-value**	**Proportion**	**Result**
Frequency	0.657933	56/56	Pass
Block frequency	0.455937	56/56	Pass
Runs	0.739918	55/56	Pass
Longest runs of ones	0.137282	55/56	Pass
Cumulative sums	0.739918	56/56	Pass
Rank*	0.669313	56/56	Pass
Discrete Fourier transform	0.005762	56/56	Pass
Approximate entropy	0.657933	56/56	Pass
Serial	0.289667	56/56	Pass

Microsynth synthesis 1, mapping scheme C (A→ 0, C → 1, T → 1, G → 0).

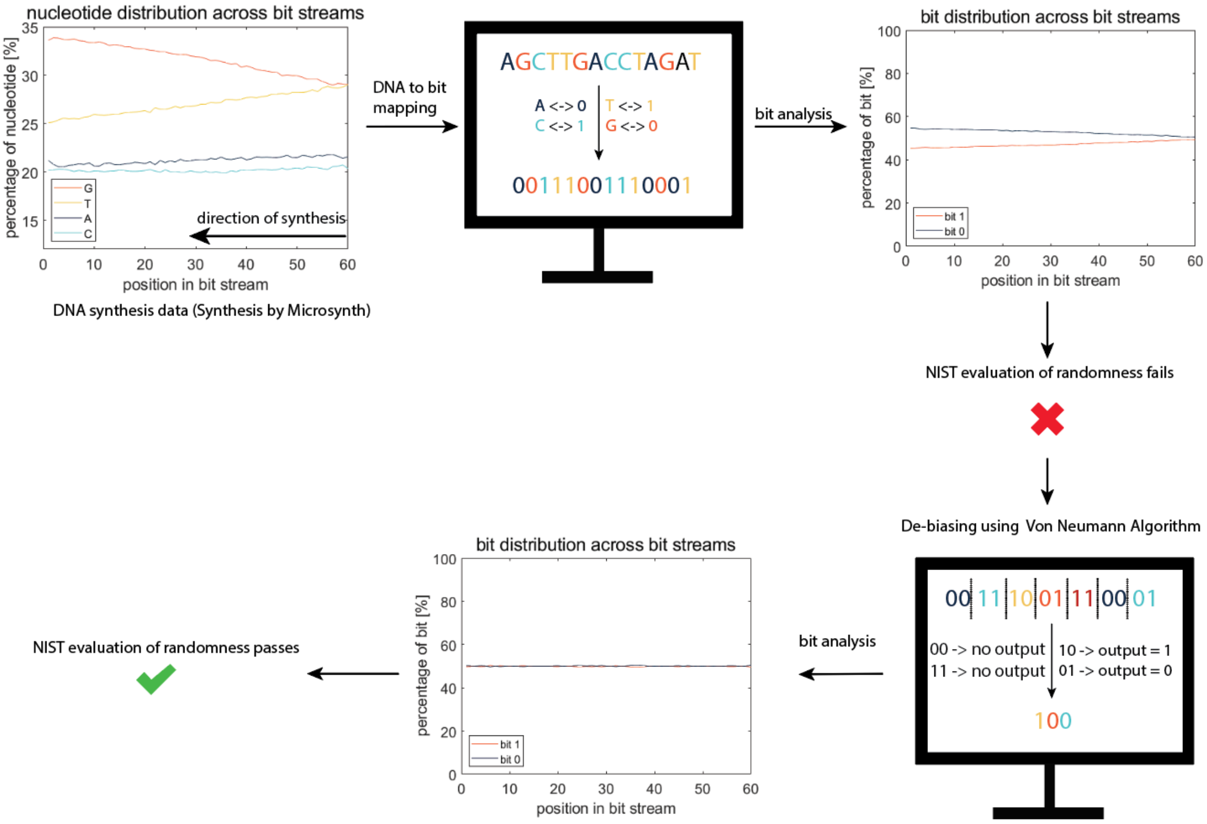

Supplementary Figure 2d: Microsynth synthesis 1, mapping scheme C (A→ 0, C → 1, T → 1, G → 0)*. Data analysis procedure showing de-biasing effect of the Von Neumann algorithm.*

Supplementary Table 8: NIST statistical test evaluation of Microsynth synthesis 1, mapping scheme C (A→ 0, C → 1, T → 1, G → 0), before de-biasing.

**NIST statistical test**<br>**Before de-biasing**	**P-value**	**Proportion**	**Result**
Frequency	0	42/56	Fail
Block frequency	0	52/56	Fail
Runs	0.096578	50/56	Fail
Longest runs of ones	0	48/56	Fail
Cumulative sums	0	45/56	Fail
Rank*	0.574903	56/56	Pass
Discrete Fourier transform	0.040108	51/56	Fail
Approximate entropy	0.075719	52/56	Pass
Serial	0.137282	55/56	Pass

Supplementary Table 9: NIST statistical test evaluation of Microsynth synthesis 1, mapping scheme C (A→ 0, C → 1, T → 1, G → 0), after de-biasing.

**NIST statistical test**<br>**After de-biasing**	**P-value**	**Proportion**	**Result**
Frequency	0.191687	56/56	Pass
Block frequency	0.739918	56/56	Pass
Runs	0.096578	56/56	Pass
Longest runs of ones	0.971699	55/56	Pass
Cumulative sums	0.122325	56/56	Pass
Rank*	0.191687	56/56	Pass
Discrete Fourier transform	0.153763	56/56	Pass
Approximate entropy	0.779188	55/56	Pass
Serial	0.455937	56/56	Pass

Microsynth synthesis 1, mapping scheme D (double mapping scheme A→ 00, C → 11, T → 10, G → 01).

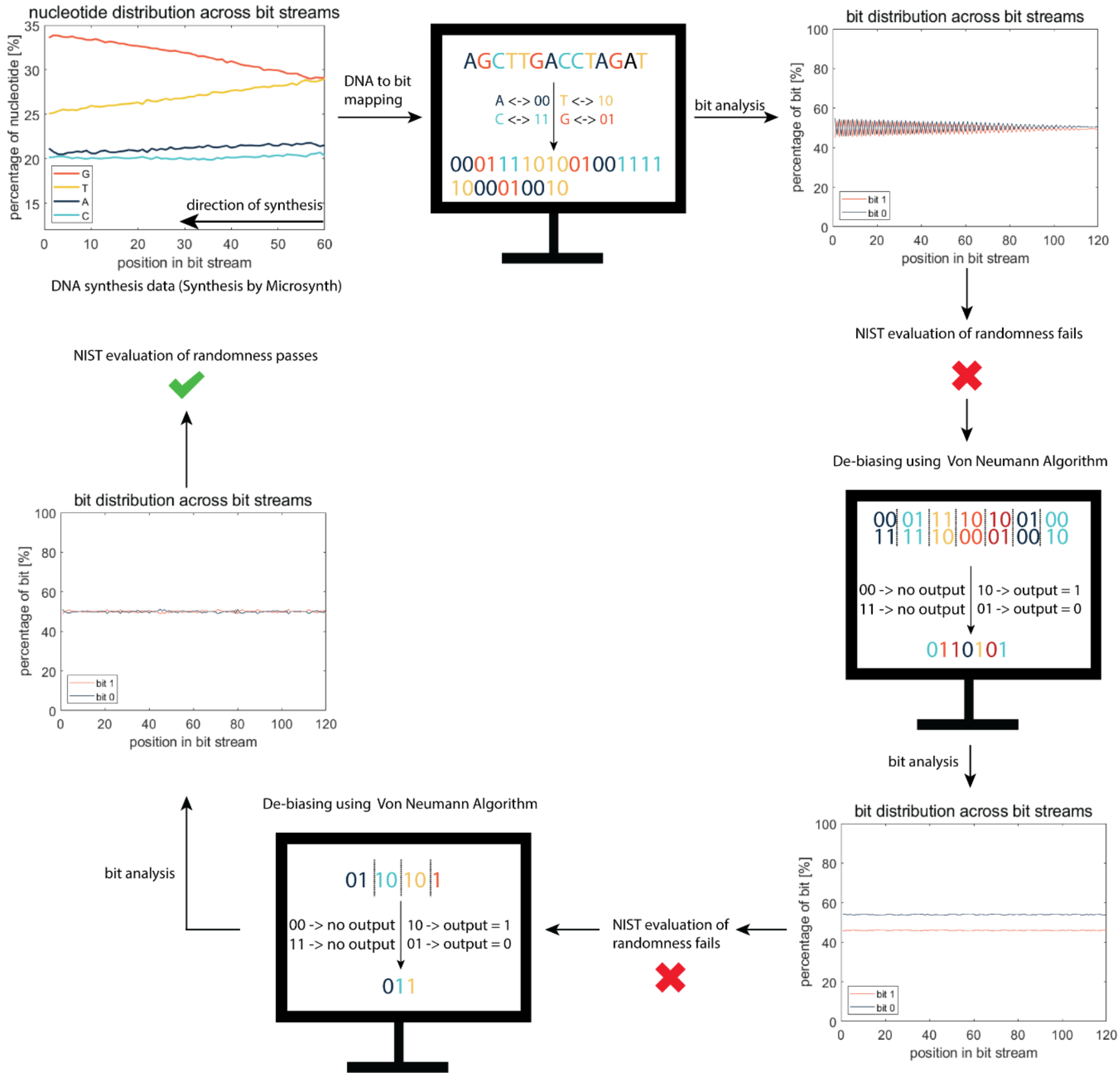

Supplementary Figure 2e: Microsynth synthesis 1, mapping scheme D (double mapping scheme A→ 00, C → 11, T → 10, G → 01). *Data analysis procedure showing de-biasing effect of the Von Neumann algorithm.*

Supplementary Table 10: NIST statistical test evaluation of Microsynth synthesis 1, mapping scheme D (double mapping scheme A→ 00, C → 11, T → 10, G → 01), before de-biasing.

**NIST statistical test**<br>**Before de-biasing**	**P-value**	**Proportion**	**Result**
Frequency	0.534146	56/56	Pass
Block frequency	0.419021	56/56	Pass
Runs	0.000000	13/56	Fail
Longest runs of ones	0.000000	46/56	Fail
Cumulative sums	0.017912	56/56	Pass
Rank*	0.262249	56/56	Pass
Discrete Fourier transform	0.015598	55/56	Pass
Approximate entropy	0.000000	43/56	Fail
Serial	0.122325	56/56	Pass

Supplementary Table 11: NIST statistical test evaluation of Microsynth synthesis 1, mapping scheme D (double mapping scheme A→ 00, C → 11, T → 10, G → 01), after first de-biasing.

**NIST statistical test**<br>**After FIRST de-biasing**	**P-value**	**Proportion**	**Result**
Frequency	0.000000	31/56	Fail
Block frequency	0.000000	43/56	Fail
Runs	0.23681	52/56	Pass
Longest runs of ones	0.000000	44/56	Fail
Cumulative sums	0.000000	31/56	Fail
Rank*	0.045675	56/56	Pass
Discrete Fourier transform	0.003712	55/56	Pass
Approximate entropy	0.000439	55/56	Pass
Serial	0.455937	55/56	Pass

Supplementary Table 12: NIST statistical test evaluation of Microsynth synthesis 1, mapping scheme D (double mapping scheme A→ 00, C → 11, T → 10, G → 01), after second de-biasing.

**NIST statistical test After SECOND de-biasing**	**P-value**	**Proportion**	**Result**
Frequency	0.419021	55/56	Pass
Block frequency	0.779188	55/56	Pass
Runs	0.494392	56/56	Pass
Longest runs of ones	0.4149021	55/56	Pass
Cumulative sums	0.935716	55/56	Pass
Rank*	0.262249	54/56	Pass
Discrete Fourier transform	0.000105	55/56	Pass
Approximate entropy	0.289667	55/56	Pass
Serial	0.574903	56/56	Pass

Supplementary Information 6: Von Neumann bit stream processing for visual analysis

Supplementary to Figure 5 of the main text, the scheme depicted here, shows how bit streams were prepared for analysis

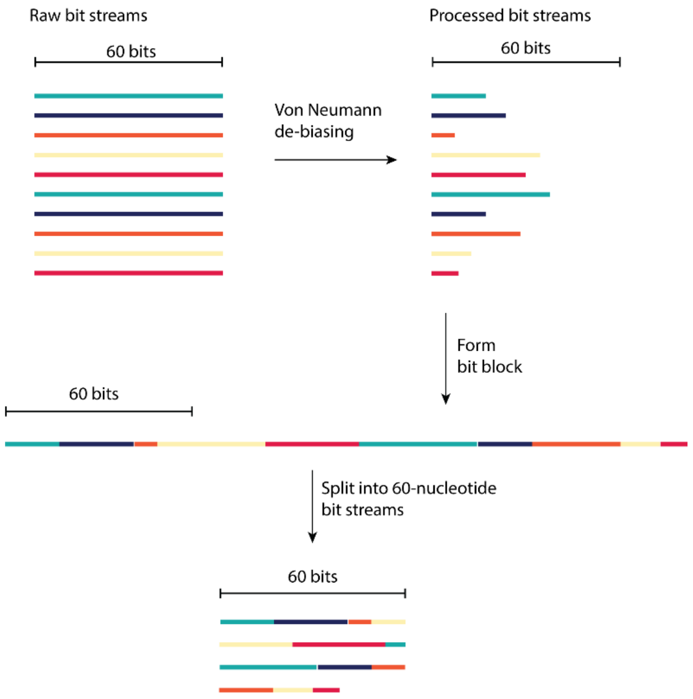

Supplementary Figure 3: Bit stream processing. *Bit stream processing for comparative analysis.*

Supplementary Information 7: List of primers used

Supplementary Table 13: List of primers used for sequencing preparation.

**Primer name**	**Sequence (5' – 3')**
0F	ACA CGA CGC TCT TCC GAT CT
0R	AGA CGT GTG CTC TTC CGA TCT
1F	ACA CTC TTT CCC TAC ACG ACG CTC TTC CGA TCT
1R	GTG ACT GGA GTT CAG ACG TGT GCT CTT CCG ATC T
2FU	AAT GAT ACG GCG ACC ACC GAG ATC TAC ACT CTT TCC CTA CAC GAC GC
2RI	CAA GCA GAA GAC GGC ATA CGA GAT **TCA AGT** GTG ACT GGA GTT CAG ACG TGT

Supplementary Information 8: Bit stream analyses for different nucleotide to bit mapping schemes

Cumulative sum of raw and processed bit stream pools (before Von Neumann de-biasing and after Von Neumann de-biasing, respectively). Figure (a) shows Microsynth synthesis 1 with mapping A (A→ 0, C → 0, T → 1, G → 1), figure (b) shows Microsynth synthesis 2 with mapping A, and figure (c) shows Eurofins Genomics Synthesis with mapping A as well. Figures (d), (e), and (f) show Microsynth synthesis 1 with mapping B (A→ 0, C → 1, T → 0, G → 1), C (A→ 0, C → 1, T → 1, G → 0), and D (A→ 00, C → 11, T → 10, G → 01), respectively. Raw bit streams are all of length 60 nucleotides, whereas processed (Von Neumann de-biased) bit streams are all shorter than 60 nucleotides, which is why the plotted distribution is narrower. For mapping D (figure (f)), the effect before and after the first Von Neumann de-biasing step is shown.

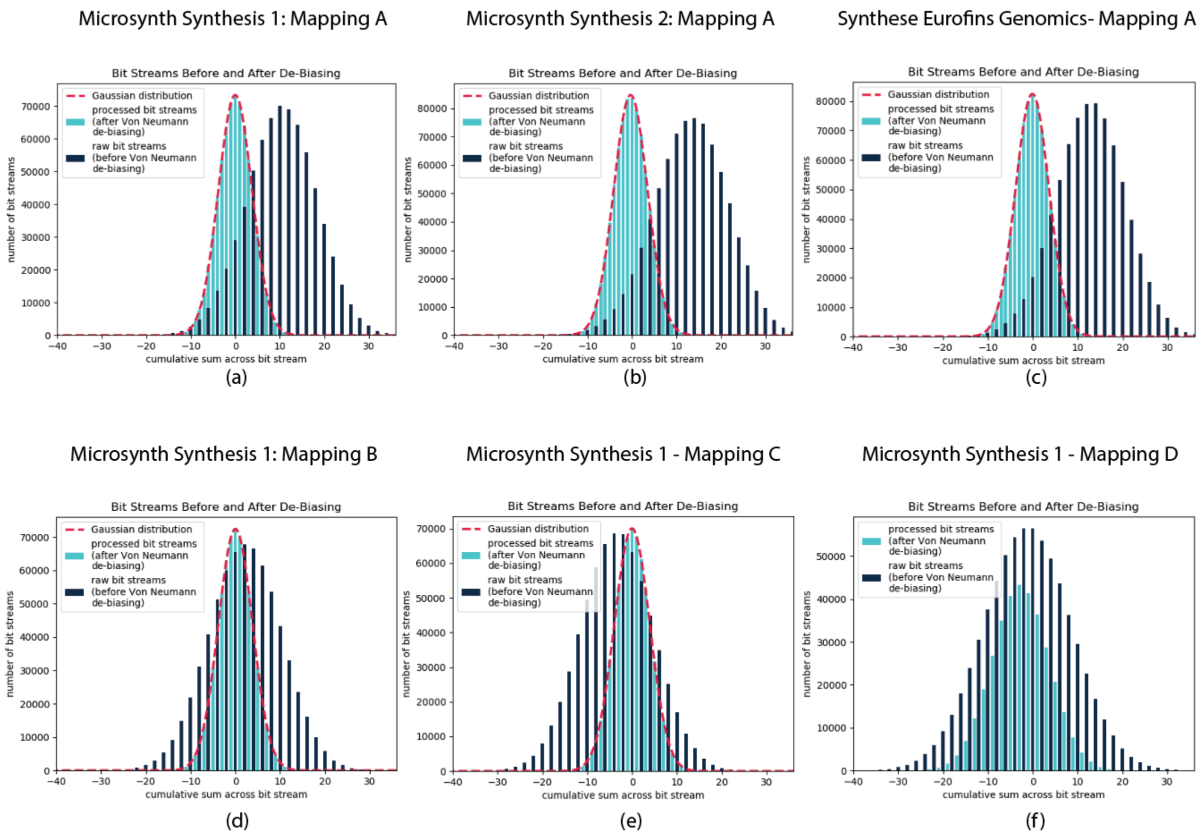

Supplementary Figure 4: Bit stream analyses*. Cumulative sum of raw and processed bit stream pools (before Von Neumann de-biasing and after Von Neumann de-biasing, respectively)*
